# Chiral germanium micro-gears for tuning orbital angular momentum

**DOI:** 10.1038/s41598-022-11245-1

**Published:** 2022-05-06

**Authors:** Abdelrahman Zaher Al-Attili, Daniel Burt, Zuo Li, Naoki Higashitarumizu, Frederic Gardes, Yasuhiko Ishikawa, Shinichi Saito

**Affiliations:** 1 Sustainable Electronic Technologies, Electronics and Computer Science, University of Southampton, Southampton, SO17 1BJ UK; 2 Electrical Engineering Department, School of Engineering Technology, Al Hussein Technical University, 11831 Amman, Jordan; 3 Department of Materials Engineering, University of Tokyo, 7-3-1 Hongo, Bunkyo-Ku, Tokyo, 113-8656 Japan; 4 Department of Electrical and Electronic Information Engineering, Toyohashi University of Technology, 1-1 Hibarigaoka, Tempaku, Toyohashi, 441-8580 Japan

**Keywords:** Microresonators, Silicon photonics, Nanophotonics and plasmonics

## Abstract

Group IV light sources with vertical emission and non-zero orbital-angular momentum (OAM) promise to unlock many novel applications. In this report, we demonstrate cylindrically symmetrical germanium micro-gear cavities, fabricated by etching a grating around the circumference of standard micro-disks, with periods ranging from 14 to 22. Photoluminescence (PL) measurements were done to identify the confined whispering-gallery modes (WGM). Finite-difference time-domain (FDTD) simulations were conducted to map the resonant modes to their modal profiles and characteristics. Vertical emission of WGMs with non-zero OAM was demonstrated, with a clear dependence of the OAM order ($\ell$) on the WGM azimuthal order and the number of micro-gear grating periods. As the chirality, or the direction of rotation, is not controlled in a symmetrical cavity, we propose introducing staircase or triangular-shaped gear periods resulting in an asymmetry. By choosing the diameter, number of periods, and the asymmetrical direction of the gear-teeth, it is possible to generate OAM signals with certain wavelength, OAM order and chirality.

## Introduction

Asymmetry was the key to innovating many photonic and electronic devices with unique characteristics^1–3^. Electronic devices such as tunneling^1^ and hetero-junction^4^ diodes, for instance, are based on asymmetrical energy-bands or asymmetrical stack of materials. Complex optical phenomena can be observed in asymmetric waveguides and cavities^5^, for a range of applications such as sensing and lasing^6^. In fact, practical asymmetric optical devices such as high-efficiency multiple quantum-well (MQW) laser diodes and vertical-external-cavity surface-emitting-lasers (VECSEL) have contributed to the realization of existing optical communications infrastructure. Remarkable efforts towards full integration of optical circuity on Si chips are being witnessed, moving from on-edge electro-optical modulation-demodulation stages, to active-optical cables, and eventually targeting all-optical on board optical interconnects^7^. On the road-map to this target, special optical components are being innovated to pave the way, such as special on-board waveguides^8^, optical multiplexers, and optical fiber-to-chip adapters^7^. More importantly, realizing complementary metal-oxide-semiconductor (CMOS)-compatible light sources^9,10^ with the ability to exchange optical data vertically is required, as flip-chip bonding is probably inevitable to realize such an optical data link between CMOS chips and printed boards^7^. This vertical communication link can be achieved by a dedicated coupling device after a standard horizontally emitting light source. Adiabatic couplers^11^, micro-electromechanical systems (MEMS) mirrors, and grating couplers are examples of such components. A more efficient way, however, would be realizing a CMOS light source with vertical emission capability. Although VECSEL structures are an option, yet there is a room for novel cavities to compete in terms of fabrication and yield^12,13^.

Vertical emission has been demonstrated from much simpler CMOS-compatible cavities based on germanium (Ge) micro-disks with a grating imposed around the circumference to interact with the confined whispering-gallery modes (WGM)^12^. Such cavities can be micro-disks or rings with a grating etched or deposited on top^13^, a ring with a grating etched on the inner circumference^14^, or a micro-disk with a grating on the outer circumference^12^. The latter form is referred to as a micro-gear cavity^12,15,16^, having privileges in terms of simpler fabrication as the grating is made from the same disk material, and easier electrical and optical pumping compared to ring cavities. Interestingly, vertically-emitted modes out of micro-gears can have non-zero orbital-angular momentum (OAM) due to the rotating nature of WGMs. Which means these modes rotate around the vertical axis of propagation as they are emitted as a vortex of light^12^. Although for some practical applications, such as optical tweezers, the order and chirality being clockwise (CW) or counter clockwise (CCW) of the OAM modes is not critical, yet, for more complex computational or communications functionalities, the order and the chirality of the OAM signal must be controlled. The OAM order is quantized, being an integer often referred to as $\ell$, describing the number of phase discontinuities (transitions from $-\pi$ to $\pi$) of the rotating mode. An OAM of 1 indicates a single transition of the rotating field’s phase from $-\pi$ to $\pi$ with a phase singularity in the middle, resulting in a zero field intensity in the middle and two lobes of field maxima centered around it^14,17^. The chirality, on the other hand, defines the optical mode propagating as a vortex around the traveling direction being CW or CCW. Controlling the chirality is a challenge in the case of WGMs, as it requires defining the direction of rotation of the WGMs within the micro-cavity. Passive control of OAM chirality is possible using complex designs such as spirals^18,19^, or plasmonic-photonic techniques^20^. More recently demonstrations of active control of chirality were also reported^21^.

**Figure 1:**
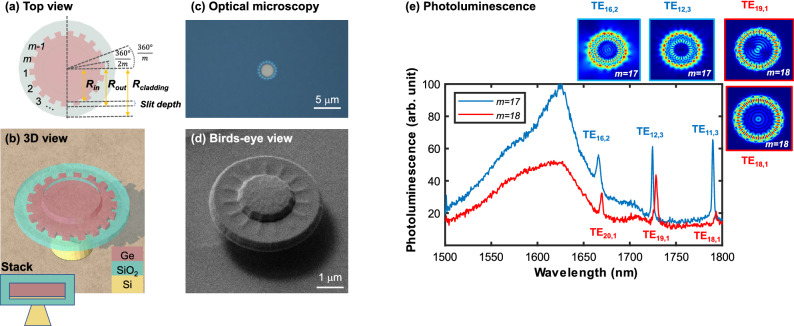
Micro-gear cavity: (**a**) Design parameters including the number of grating periods (*m*), and the slit-depth ($R_{out}$–$R_{in}$). (**b**) 3D view of a germanium micro-gear cavity with $\hbox {SiO}_{{2}}$ cladding on a Si pedestal, the inset on the bottom left corner represents the stack. (**c**) Optical microscopy image of a micro-gear after etching. (**d**) Birds-eye view using focused-ion beam (FIB) microscope of a fabricated device. (**e**) Photoluminescence spectra of micro-gears of 17 and 18 periods with sharp-peak whispering-gallery modes. The surface plots show the simulated mode profiles represented by the magnitude of the electric field captured at the top surface of the micro-gear as annotated, with blue (red) annotations are used for $m=17$ ($m=18$) modes.

In this paper, we investigate the spectral characteristics of micro-gear cavities and their dependence on the physical gear grating periods (Fig. 1). Photoluminescence (PL) measurements confirm the confinement of WGMs of different orders within the direct band-gap of Ge micro-gears with grating periods ranging from 14 to 22. Mapping the measured resonant modes to their corresponding modal profiles using finite-difference time-domain (FDTD) simulations, the OAM order of each mode was determined and related to the WGM order and the physical gear design affected mainly by the number of periods (${ m}$ in Fig. 1a). It was observed that degenerate modes with the same non-zero OAM exist due to the random direction of WGM rotation given the azimuthal symmetry of a micro-gear cavity. Accordingly, we propose a simple solution to control the direction of rotation of the WGMs within micro-gears, thus defining the OAM chirality to be CW or CCW, by introducing asymmetry to the gear-tooth shape. The standard symmetric micro-gear period which consists of a Ge section and an etched section with similar angular spans is replaced with a 4-segment period with a different radius assigned to each segment, resulting in a staircase or a triangular-shaped gear-tooth. FDTD simulations confirm a certain directional preference of WGMs corresponding to the asymmetrical design of the cavity, resulting in a defined OAM rotational direction. Controlled OAM signals with orders up to 8 and determined chirality can be obtained by tuning the micro-gear design parameters.

## Results

Emission of the cavity provide the privilege of inspecting the spectral response from an internal source, overcoming the issues of coupling to an external waveguide and the resulting irrelevant resonant modes. Ge^9,10^ and $\hbox {Ge}_{{x}} \hbox {Sn}_{1-x}$^22–24^ alloys are being investigated as candidate materials for CMOS-compatible light sources. Thus, in our study we have integrated our design on a Ge micro-gear cavity (Fig. 1a–d), while the cavity design itself is applicable to any other waveguiding material. Subsections below summarize the observations of PL measurements, followed by a detailed analysis using FDTD simulations.

### Photoluminescence measurements

PL measurements were conducted at room-temperature such that pumping, and signal collection was done from the above. Fig. 1d shows a bird’s-eye view of a fabricated micro-gear device with symmetric periods as seen using a focused-ion beam (FIB) microscope. Devices with grating periods (${ m}$) ranging from 14 to 22 were examined. Figure 1e plots the PL spectra of micro-gears with periods (${ m}$) equal to 17 and 18. The emission peak corresponds to the direct band-gap energy of Ge^12,25–30^, with the main peak around 1630 nm representing the $\Gamma$ to heavy-hole (HH) transition, while the higher wavelengths are due to $\Gamma$ to light-hole (LH) transition^12,25–30^. A slight red-shift in the Ge direct band-gap peak is consistent with a biaxial tensile-strain value of $\approx$ 0.4% imposed on the micro-gears by the surrounding silicon dioxide^30^. This tensile strain value was estimated using Raman spectroscopy^12,28^. The spectra confirm the confinement of WGM resonances within the micro-gears of even and odd-numbered grating periods. The WGMs of the $m=18$ micro-gear were identified as $\hbox {TE}_{20,1}$, $\hbox {TE}_{19,1}$, and $\hbox {TE}_{18,1}$, occurring at 1670, 1727, and 1792 nm, respectively. Where TE indicates that the electric field is confined in-plane with the cavity. The subscript numbers represent the azimuthal and radial orders of the WGMs, respectively. Azimuthal order being the number of full wavelengths around the circumference, while the radial order is the number of field peaks along the radius of the cavity. ${ m}$ = 17 micro-gear WGMs were identified as $\hbox {TE}_{16,2}$, $\hbox {TE}_{12,3}$, and $\hbox {TE}_{11,3}$, at 1666, 1724, and 1789 nm, respectively, and are slightly blue-shifted compared to the ${ m}$ = 18 modes. Relating this blue-shift to a possible change in the effective refractive index imposed by the different number of grating periods is not straightforward, because the WGM orders are not the same in both cavities.

**Figure 2:**
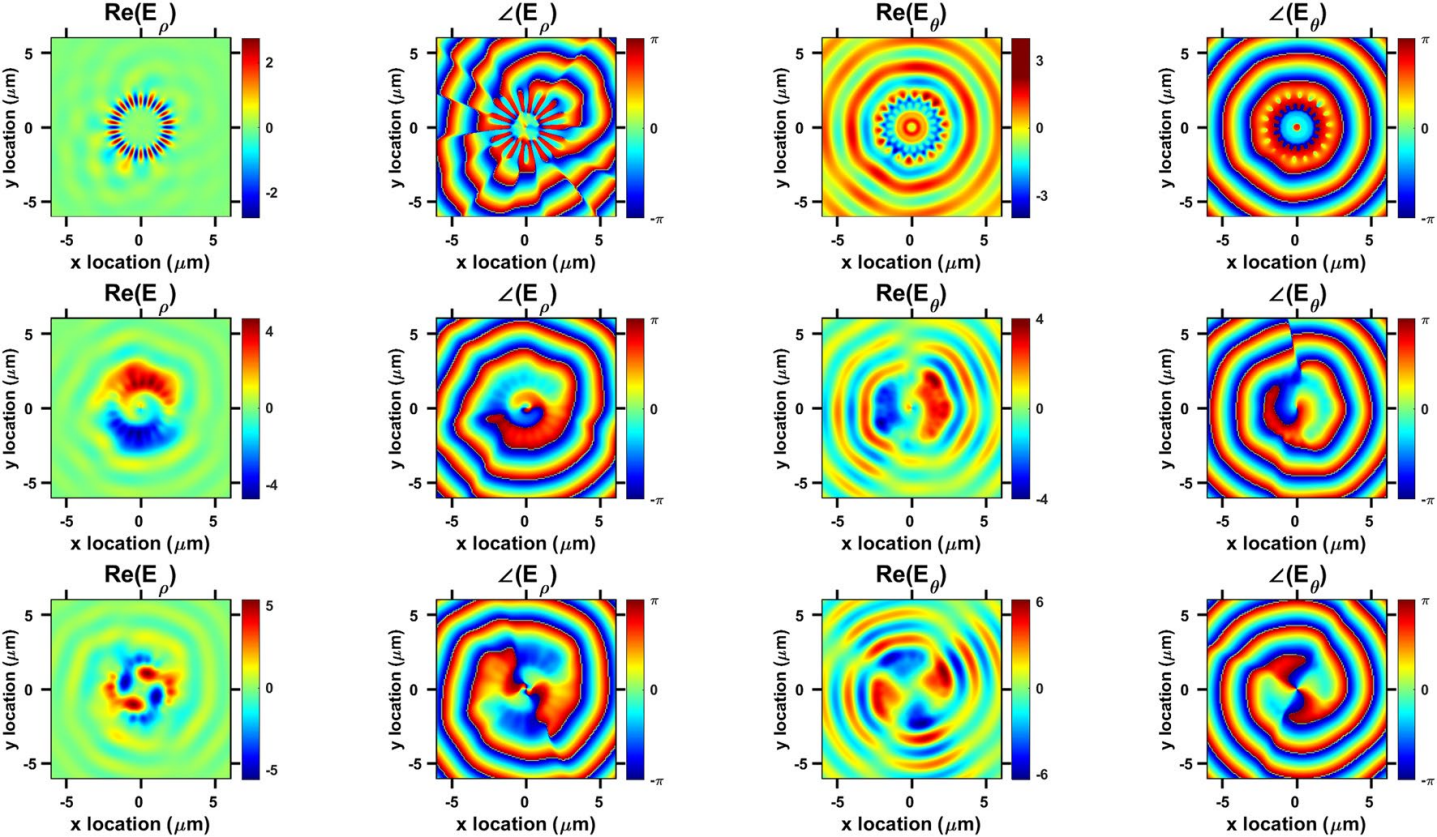
Finite-difference time-domain (FDTD) simulations of in-plane electric field components $\hbox {E}_{\rho }$ and $\hbox {E}_{\theta }$ captured at 0.5 $\upmu$m above the $m=18$ micro-gear surface. First, second and third rows plot the fields for $\hbox {TE}_{18,1}$ (at 1792 nm), $\hbox {TE}_{19,1}$ (at 1727 nm), and $\hbox {TE}_{20,1}$ (at 1670 nm), respectively. Real values of the electric field components and the corresponding phase plots indicate OAM order of 0, 1, and 2, respectively.

**Figure 3:**
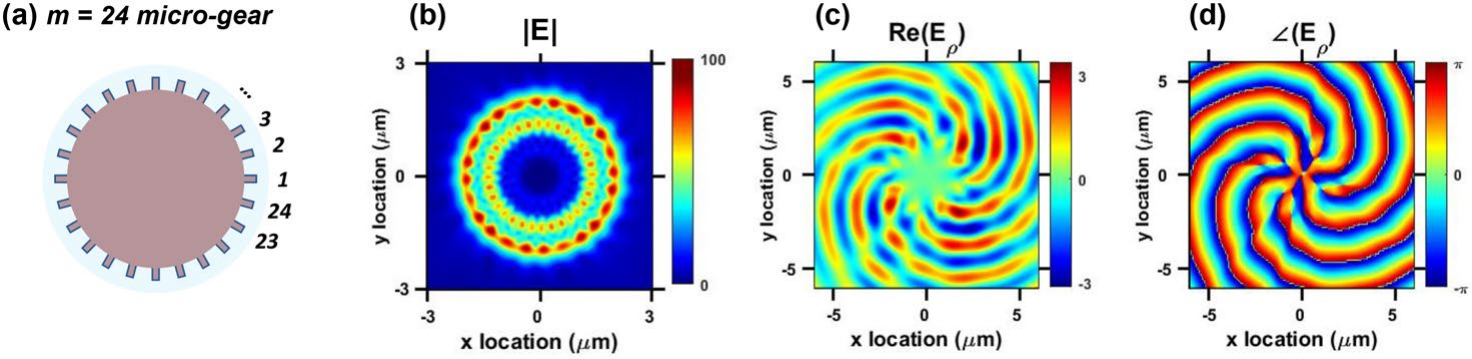
Higher-order whispering-gallery mode (WGM) example: (**a**) sketch of $m=24$ micro-gear cavity. (**b**) Magnitude of the electric field components of $\hbox {TE}_{16,2}$ (at 1675 nm) captured at the top surface of the micro-gear. (**c**) Real value of $\hbox {E}_{\theta }$ captured at 0.5 $\upmu$m above the micro-gear. (**d**) The corresponding phase plot indicates an OAM order of 8.

### OAM order

Micro-gear cavities have been used before to reduce the lasing threshold, explained mainly by the enhancement of the Purcell effect^15,16^. Recently, vertical emission of certain resonant WGMs with non-zero OAM have been demonstrated in Ge micro-gears^12^, explained by the diffraction of WGMs as they interact with the physical grating. Three-dimensional FDTD simulations were conducted to correlate the OAM order to the WGM order and the physical micro-gear design. Figure 2 shows surface plots of the in-plane electric fields and their corresponding phase distributions of the resonant WGMs of the $m=18$ micro-gear, as measured by PL in Fig. 1e. The electric field distributions plot the real value of the in-plane components $\hbox {E}_{\rho }$ (radial) and $\hbox {E}_{\theta }$ (azimuthal), in addition to their corresponding phases. The helical distributions of the field and phase plots of $\hbox {TE}_{19,1}$ and $\hbox {TE}_{20,1}$, with the phase singularity resulting in a zero-field value in the middle, is a main characteristic of non-zero OAM fields. Two (four) field intensity lobes with corresponding single (double) phase transitions from $-\pi$ to $\pi$ indicate an OAM order of $\pm 1$ ($\pm 2$), respectively. The ± sign accounts for the random chirality, or direction of rotation, of the fields as the WGM propagation direction is random within the cavity due to the cylindrical symmetry. It is also evident that a phase shift of $\pi /2$ exists between the two in-plane field components. This helical field behavior is absent in $\hbox {TE}_{18,1}$ (OAM = 0). Mapping the modal profiles to the WGM orders and the number of the physical gratings, it is found that the OAM order is defined as the integer difference between the WGM azimuthal number and the number of the micro-gear periods, consistent with the previous findings reported in literature^14^. For instance, $\hbox {TE}_{19,1}$ in the $m=18$ micro-gear has an OAM order of 19-18 (= $\pm 1$) and so on, as confirmed in micro-gears with different periods (*m*). Higher order OAM values could be obtained by changing the design such as the mode shown in Fig. 3 corresponding to a micro-gear with 24 grating periods ($m=24$, Fig. 3a) and a confined WGM $\hbox {TE}_{16,2}$ (Fig. 3b). The resulting mode propagating upwards from the cavity has an OAM order of $\pm 8$ (24-16), as evident in the field and phase distributions (Fig. 3c,d). It is worth of mentioning that the radial order of the WGM is irrelevant to the resulting OAM order in this case, as the two field maxima along the radial direction have not affected it. In all the modes presented in Figs. 2 and 3, the chirality or the direction of rotation was not controlled, which is unsuitable for certain applications.

**Figure 4:**
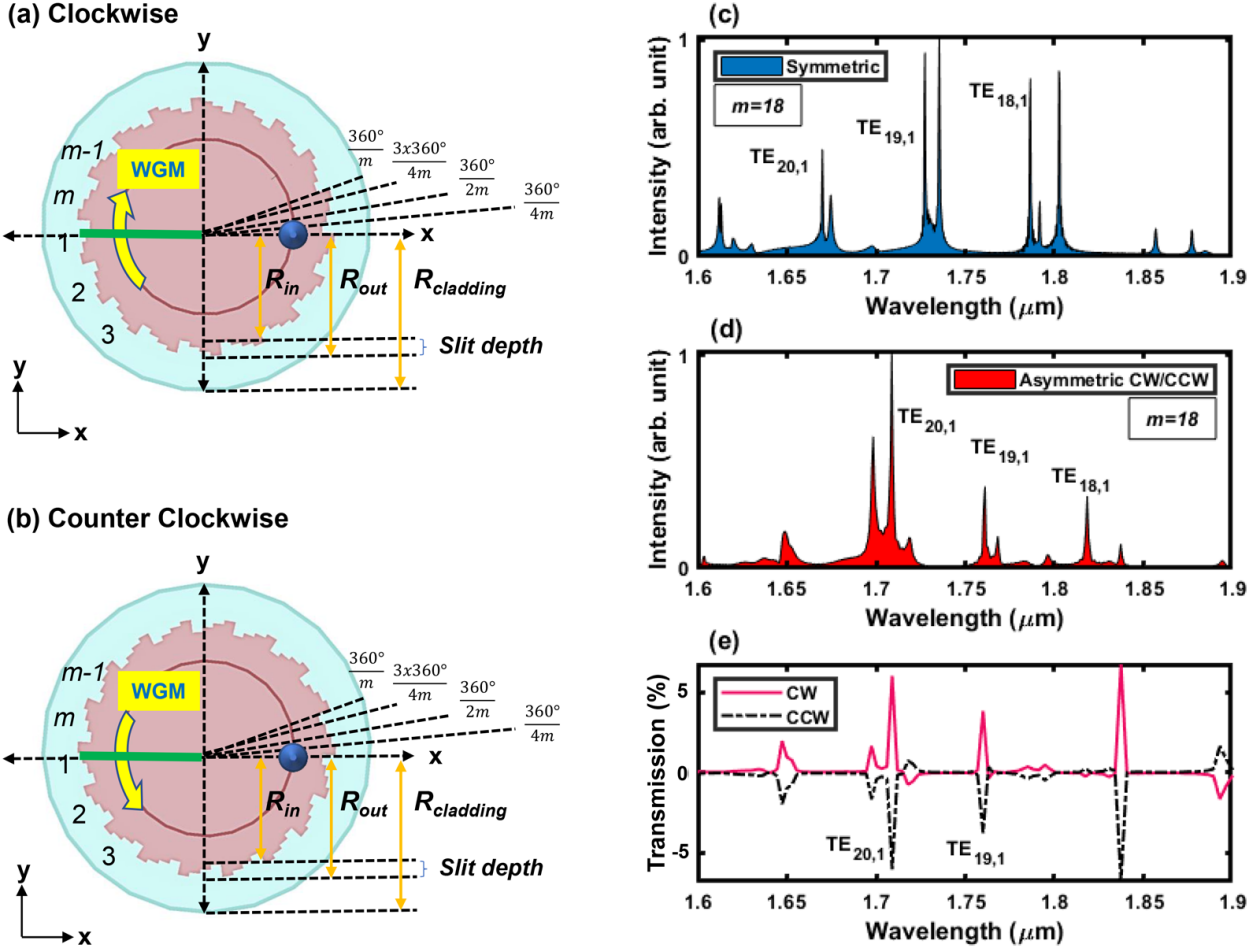
Asymmetric micro-gear cavities promoting (**a**) clockwise, and (**b**) counter clockwise whispering-gallery modes (WGM) rotation. Each gear-tooth is divided into 4 segments with a different radius resulting in staircase or triangular-shaped periods. (**c**) Simulated spectrum of a symmetric micro-gear as shown in Fig. 1a, $m=18$. (**d**) Simulated spectrum of CW or CCW asymmetric micro-gear cavities with 18 periods ($m=18$). (**e**) Simulated transmission of the WGMs in CW and CCW asymmetric micro-gears ($m=18$) captured through the cross-section of the cavity as they pass at the location of the yellow arrow in (**a**,**b**). Transmission is defined as the normalized power flow through the cross-sectional area of the cavity through a monitor that is placed at the position marked by the solid green line in (**a**,**b**) extending from *x* = − 2.25 to 0 $\upmu$m, and *y* = 0 $\upmu$m. Positive (negative) transmission sign means a power flow towards the positive (negative) y-axis through the monitor surface area. The fields are equal in magnitude but opposite in direction confirming reversing the WGM propagation by asymmetry. The excitation source is placed at the position marked by the blue circle on the positive x axis (at $y = 0$ and $x = R_{in}-100$ nm).

### OAM chirality

**Figure 5:**
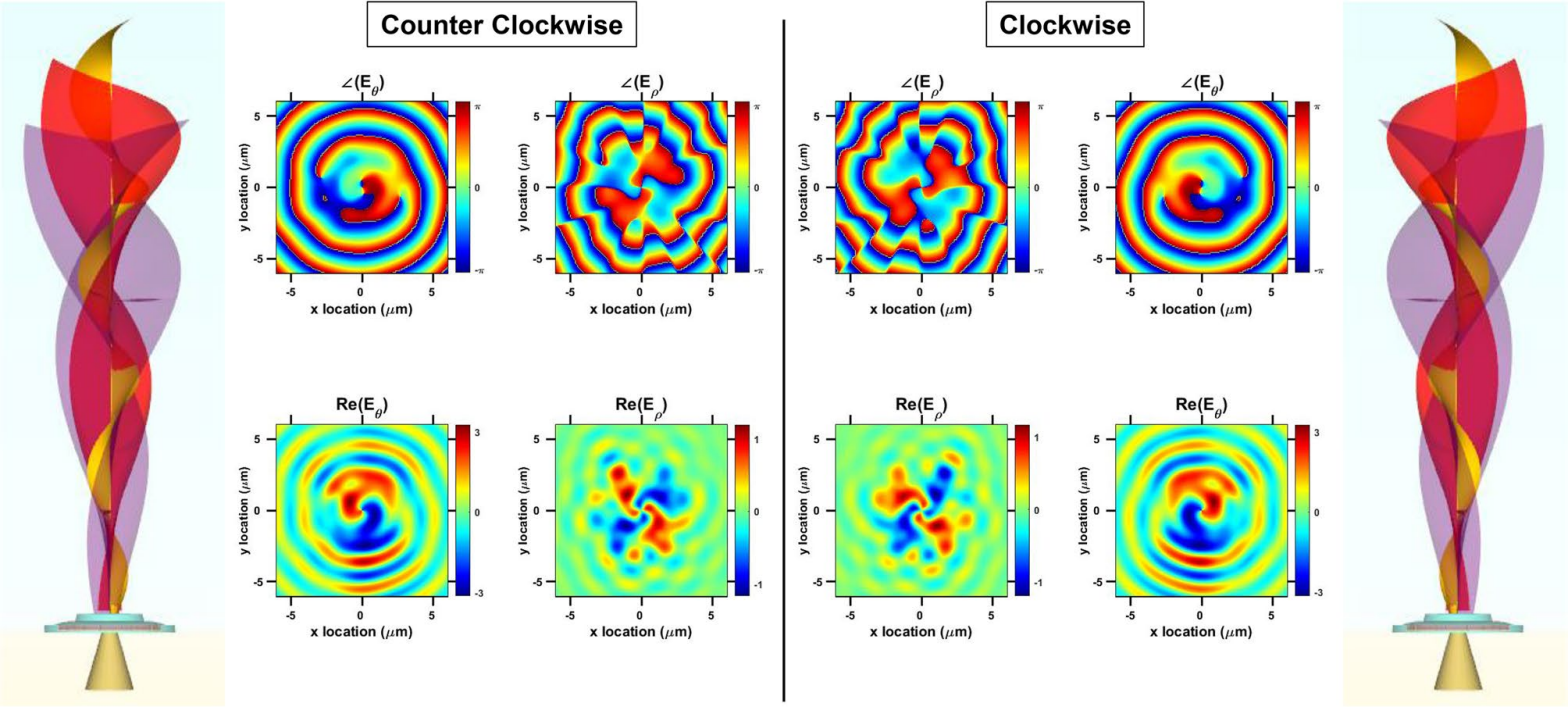
Controllable OAM order and chirality: a sketch representing the expected emission of clockwise (CW) and counter clockwise (CCW) asymmetric micro-gear cavities. Surface plots of the in-plane electric field components and the corresponding phase plots confirm controlling the OAM chirality for different orders. The modes with similar OAM order in CW and CCW cavities correspond to the same WGMs at the same wavelengths. Outermost sub-figures plot $\ell = 1$ modes at 1.761 $\upmu$m, the innermost sub-figures plot $\ell = 2$ modes at 1.709 $\upmu$m. The OAM order is defined by the WGM azimuthal order and the number of the micro-gear periods. The OAM chirality is reversed for the exact same modes at the same wavelengths depending on the asymmetrical direction of the cavity.

Cylindrical symmetry imposes equal probabilities on the direction of rotation for the confined WGMs, either being CW or CCW, so is the corresponding chirality of the vertically-emitted modes with non-zero OAM. For example $\hbox {TE}_{19,1}$ at 1.727 $\upmu{\text{m}}$ (Fig. 4c) can rotate CW or CCW due to azimuthal symmetry. We propose imposing asymmetry to the gear-tooth shape to enforce a preference onto the WGM rotation direction. Figures 4a,b show the top view of the modified micro-gear cavities. Every grating period is divided into four segments, each segment having a certain radius value resulting in a staircase or a triangular-shaped gear-tooth. If the micro-gear cavity has *m* periods, then each period has an angular span of $360^{\circ }/m$. This period is further divided into four circular sectors extending along angular spans of $360^{\circ }/4m$, $360^{\circ }/2m$, $3\times 360^{\circ }/4m$, and $360^{\circ }/m$, or simply, each of the four segments has an angular span of $360^{\circ }/4m$. In our design we assumed that each segment extends 50 nm along the radial direction beyond the previous segment, or the new slit-depth is divided equally across the four-segments. For instance, if the inner radius of the cavity ($R_{in}$) is $2 \,\upmu$m, then the first $360^{\circ }/4m$ sector will have a radius of 2 $\upmu$m, the following three sectors each having an angular span of $360^{\circ }/4m$ will have radius values of 2.05, 2.10, and 2.15 $\upmu$m, respectively. The resulting triangular-shaped period is then repeated *m* times across the circumference of the cavity. As indicated in Fig. 4, WGM propagation direction will accordingly have a preference of CW or CCW. It can be seen as if the WGM prefers going in the same direction with the arrow-like shape of the triangular-shaped periods. Figure 4c,d compare the spectral response of the symmetric (Fig. 1) and asymmetric (Fig. 4) micro-gear cavities with 18 periods ($m=18$), respectively. First order WGMs are annotated for both types of cavities as $\hbox {TE}_{20,1}$, $\hbox {TE}_{19,1}$, and $\hbox {TE}_{18,1}$. These modes have the best confinement in the case of $m=18$ micro-gears as discussed in the PL section (Fig. 1e). The spectra of CW and CCW asymmetric micro-gears are the same (Fig. 4d), hence only one of them is shown. Comparing the spectrum of symmetric and asymmetric micro-gears, it is found that WGMs of the same order occur at higher wavelengths in the case of asymmetric micro-gears, for example, $\hbox {TE}_{19,1}$ ($\hbox {TE}_{20,1}$) is located at 1.761 $\upmu$m (1.709 $\upmu$m) in the asymmetric micro-gear, compared to 1.727 $\upmu$m (1.67 $\upmu$m) in the symmetric cavity, indicating a red-shift of approximately 40 nm. This can be explained by the higher effective refractive index observed by the WGMs in the asymmetric cavity as the grating periods have a larger slit depth. Symmetric cavities have a total slit depth of 50 nm, while the asymmetric ones have 50 nm for each segment within the period. This can also be seen as a larger effective radius of the asymmetric cavity, resulting in the confinement of higher wavelength modes. More importantly, it can be observed that some modes are filtered out in the case of the asymmetric cavities, with higher confinement provided for WGMs propagating in the direction of preference. In other words, CW and CCW rotating WGMs would still exist within the asymmetric cavity, yet one of them is promoted due to asymmetry. For instance, in an asymmetric CW micro-gear (Fig. 4a), the CW WGM rotation direction has higher probability, or better quality factor, as the other path (CCW) is more lossy. This can be also viewed as a difference in the effective refractive index observed by the WGMs through different rotational directions. As an example, consider the same WGM, $\hbox {TE}_{19,1}$ at 1.761 $\upmu$m (Fig. 4d), the simulated direction of rotation is CW, as shown in Fig. 4e. Figure 4e measures the transmission of the WGMs, or the power flow through the cross-section of the cavity normalized to the source power used in simulations. Positive transmission indicates a power flow towards the positive *y* axis and vice versa. The resonant modes were recorded as they pass the cross-section of the cavity along the negative side of the *x*-axis, and *y* = 0 $\upmu$m (at the location of the solid green line in Fig. 4a,b). Notice that the exact same mode, at the exact same wavelength, has the same transmission value but the opposite transmission sign (direction) in the case of a CCW cavity (Fig. 4b), as shown in Fig. 4e. Evidently, the intensity of the resonant WGMs is the same in CW and CCW asymmetric cavities, but the direction is clearly reversed. Remarkably, $\hbox {TE}_{18,1}$ mode has no CW or CCW preference, coinciding with the previous result indicating a zero OAM for this mode in an 18-period micro-gear. Moreover, Fig. 4e indicates that some fields opposite to the direction of preference might exist, for example, the mode at 1.718 $\upmu$m propagates CCW in a CW cavity, and vice versa. Yet, such modes seem to be attenuated compared to the modes propagating with the direction of preference defined by the asymmetry. The discretely segmented structure of the gear-tooth provides a room for optimization to increase the contrast ratio between the two WGM directions, in addition to other characteristics such as the quality factor. Field and phase profiles of $\hbox {TE}_{20,1}$ and $\hbox {TE}_{19,1}$ WGMs (simulated in Fig. 4d,e) in the asymmetric $m=18$ CW and CCW cavities are plotted in Fig. 5. These modes are located at the exact same wavelengths in both CW and CCW asymmetric cavities, as defined above. The modes are captured at $\approx 1 \,\upmu$m above the cavity’s surface as they propagate vertically. The mode order is still defined by the difference between the asymmetric micro-gear grating periods ($m=18$) and the azimuthal WGM order, resulting in $\ell = 2$ and $\ell = 1$ for $\hbox {TE}_{20,1}$ and $\hbox {TE}_{19,1}$, respectively. The chirality, however, is obviously reversed for the same modes in CW (Fig. 4a) and CCW (Fig. 4b) cavities.

## Discussion

Simple cavities providing vertically-emitted modes with non-zero OAM of defined order and chirality pave the way towards multiple novel applications, especially if realized on a Si platform^7^. Namely, on-chip optical communications with two-dimensions of multiplexing—wave and space division multiplexing—for extremely high data-rates^31^. Consequently, Ge was chosen being a promising CMOS-compatible gain medium^9,10,25–29^, especially with the recent lasing reports of Ge and $\hbox {Ge}_{{x}} \hbox {Sn}_{1-x}$^22–24^, although the cavity concept applies to any waveguiding material. This is evident in the sharp-peak resonances observed in Fig. 1e considering that the measurement was conducted at room temperature. Design parameters such as the number of periods affect the quality of the modes significantly. For instance, the cavity with 17 periods resulted in finer modes, or lower optical loss, compared to the 18 periods. This can be attributed to mode filtering due to the miss-match^15,16^ between the odd-numbered physical grating periods (${ m}$=17) and the periods of the first-order WGMs that are confined in the micro-gear with even periods (${ m}$=18). This mode filtering reduces the number of resonances within the gain region resulting in finer modes. Moreover, higher-order WGMs are confined in the odd-period cavity, as shown in the surface plots in Fig. 1e. Higher-order WGMs have a considerable amount of their electric field intensity squeezed away from the outer circumference of the cavity towards the middle, resulting in less scattering losses. Moreover, the outer circumference of the micro-cavities is expected to be less tensile-strained compared to the inner regions^12,32^, thus providing lower optical gain.

Conventional micro-disk cavities emit WGMs radially or in-plane without a directional preference^33^. Several modified micro-cavities were reported to enhance the emission in a certain direction, with modifications that can be categorized into three main concepts: deforming the micro-disk, adding defects, notches, or particles on the micro-disk, and etching a grating around the whole cavity. For instance, deformation of micro-disks into a disk with a flat side^34,35^, ellipse^36^, stadium-shaped^37^, triangle with rounded edges^38^,and Limacon-shaped micro-cavities^39^ were reported resulting in an in-plane unidirectional emission. Adding a defect such as nano-particles above or near the cavity^40^, etched defects^41^, or a notch^41^ also result in an enhanced in-plane unidirectional emission. Gratings fabricated around a micro-disk or ring, either on the outer^12^ or inner circumference^14^, or on top^13^, cause the WGMs to emit out-of-plane. The first two ideas result in an in-plane unidirectional emission, while the last one results in a vertical out-of-plane emission. Such modifications do not impose a certain rotational direction on the WGM within the cavity, they just affect the radiation direction. The asymmetric micro-gear cavities proposed in this report provide a preference for a certain rotational direction of the WGMs within the cavity, in addition to the out-of-plane unidirectional radiation, as evident in Figs. 4 and 5. Figure 4e indicates CW and CCW propagation in the cavities in (a) and (b) of the same figure. Figure 5 sketches the concept of vertical emission of WGMs with different OAM order and chirality. Vertical emission is evident in the plotted fields as they are captured above the surface of the cavity^12^. This is beneficial for multiple applications, especially for on-chip optical communications, in which data communication from chip to board is to be done vertically^7,31^. The surface plots clearly demonstrate the control of rotational direction as the lobes of the field intensities and the phases are reversed for CW and CCW plots. The modes of the same OAM order in Fig. 5 are the same WGMs occurring at the same wavelengths in CW and CCW cavities. By designing multiple cavities each with a certain diameter, number of periods (*m*), and an asymmetric gear-tooth direction, it is possible to generate specific optical modes with certain wavelength, OAM order, and chirality, which can be multiplexed spatially in a later stage^31^. Modes with different OAM will be spatially separated similar to coinciding rings with the higher OAM being the out-most ring^31,42^. At the receiving end, modes with opposite chirality can be separated as they can be deflected to opposite off-axis directions^42^.

## Methods

Micro-gears investigated in this report have an outer diameter of 4 $\upmu$m, a slit-depth of 50 nm, and periods ranging from 14 to 22. The devices as shown in Fig. 1d were fabricated on Ge on silicon (Si) on insulator (Ge-on-SOI) wafers. The stack consisted of 200-nm Ge, 20-nm Si, and 145-nm buried oxide (BOX) on bulk Si substrate. Initial cleaning of the Ge layer was done using diluted hydrochloric (HCl) and hydrofluoric (HF) acids in sequence. Patterning of the cavities was conducted using electron-beam lithography (EBL) with 4-nm beam spot-size and high-resolution 20-nm thick Hydrogen silsesquioxane (HSQ) resist. Such fine patterning parameters are required as the micro-gear teeth are of 50-nm depth only, which is a challenge especially for cavities with high number of periods. Reactive-ion etching (RIE) down to the BOX layer, followed by the HCl and HF cleaning, then immediate deposition of a capping $\hbox {SiO}_{{2}}$ layer was then performed. The capping layer was deposited using plasma-enhanced chemical-vapor deposition (PECVD) at 350 $^{\circ }C$, and was tuned to result in a thickness of 200 nm. This thickness is chosen to eventually result in a symmetric 145-nm-thick $\hbox {SiO}_{{2}}$ stack below and above the Ge micro-gear at the end of the fabrication process, which is advantageous as a symmetrical cladding resulting with a better effective refractive index compared to asymmetrical stacks^43^. The $\hbox {SiO}_{{2}}$ layer was then dry-etched into micro-disks after a second EBL patterning, encapsulating the concentric Ge micro-gears, with a radius of 400 nm beyond the micro-gear outer radius ($R_{out}+400$ nm). To under-etch the bulk Si below the micro-gear, we first dry-etched bulk Si using inductively-coupled plasma (ICP) etching with a high-selectivity etching recipe resulting in a 1 $\upmu$m-deep Si pillar, consuming approximately 50 nm of the capping PECVD $\hbox {SiO}_{{2}}$. Then a pedestal is formed by wet-etching the Si pillar using tetra-methyl-ammonium hydroxide (TMAH), resulting in a pyramidal-shaped pedestal^12^. This anisotropic step is essential to under-etch the outer edges of the cavity preventing WGM leakage to the substrate. It also has a role in imposing tensile strain on Ge to enhance the optical gain as the $\hbox {SiO}_{{2}}$ encapsulating layers expand^12,32^.

Photoluminescence measurements were conducted at room temperature using a confocal setup such that the excitation and collection is done from the above through a 50$\times$ objective lens. The pump laser power and wavelength were chosen as $\approx$ 2 mW and 785 nm, respectively. The collected signal was detected using a liquid-Nitrogen-cooled extended InGaAs detector, with an exposure time of 0.6 seconds, repeated and averaged over 150 times for each measurement. Three-dimensional FDTD simulations using Lumerical software were conducted to study the resonant spectra and modal profiles. The spatial dimensions of the FDTD simulation region were set to 12 $\upmu$m in the x and y directions, and 1.5 $\upmu$m in the z direction. A wavelength-dependent simulation mesh was used with 18 mesh points per wavelength, resulting in a minimum spatial discretization step of approximately 20 nm. The materials used were modeled as dispersive materials, and the simulation duration was set to 9000 fs. The excitation source used was a broadband magnetic dipole (point source) oriented in the *z* direction (out-of-plane to the cavity’s surface defined by *x* and *y* axes in Fig. 4), resulting in a non-directional electric field radiation in-plane. The location of this source was set to $y = 0$ and $x = R_{in}-100$ nm (blue circle on the positive x axis in Fig. 4a,b), and in the middle of the cavity in the *z* direction. The source pulse-shape is defined by a Gaussian-function with wavelengths ranging from 1.6 to 1.8 $\upmu$m representing a Ge-based source. The fields for the OAM data in Figs. 2 and 3 were captured at approximately 0.5 $\upmu$m above the cavity’s surface, and at 1 $\upmu$m in Fig. 5. Electric fields were converted from Cartesian to Polar vector forms. Confirming the in-plane propagation direction of the WGMs within the asymmetric cavities was done by placing a field monitor perpendicular to the WGMs path within the cavities, or in other words, coinciding with the cross-section of the micro-gears located at *y* = 0 $\upmu$m and *x* ranging from − 2.25 to 0 $\upmu$m (green solid line in Fig. 4a,b). This position imposes similar azimuthal distances from the excitation source. Transmission was recorded through this monitor, defined as the power flow through the monitor’s surface area normalized to the total source power used in the simulation. Positive and negative transmission values indicate an opposite direction of propagation through the monitor, with positive means a flow towards the positive *y* direction in our case (Fig. 4e). Simulations were conducted multiple times on symmetric and asymmetric micro-gears with different periods to ensure consistency raw data is available online in the (Supplementary Information) section.

## Supplementary Information

Supplementary Information.

## Data Availability

All data generated or analysed during this study are included in this published article and its supplementary information files.
